# Exploring prevention and control mechanisms of youth substance use: a case of North Wollo and Waghimra, Northeast Ethiopia

**DOI:** 10.3389/fpubh.2024.1487772

**Published:** 2024-12-10

**Authors:** Sindew Asmare Wedi, Biruk Beletew Abate, Tesfa Anmut Asres, Mohammed Aragaw Abera, Sentayehu Oljira Bekele, Ashenafi Kibret Sendekie

**Affiliations:** ^1^College of Social Sciences and Humanities, Woldia University, Woldia, Ethiopia; ^2^College of Medicine and Health Sciences, Woldia University, Woldia, Ethiopia; ^3^School of Population Health, Curtin University, Bentley, WA, Australia; ^4^College of Education and Behavioral Sciences, Woldia University, Woldia, Ethiopia; ^5^Department of Clinical Pharmacy, School of Pharmacy, College of Medicine and Health Sciences, University of Gondar, Gondar, Ethiopia; ^6^School of Pharmacy, Curtin Medical School, Faculty of Health Sciences, Curtin University, Bentley, WA, Australia

**Keywords:** substance use, substance use disorder, substance abuse, prevention, control, Ethiopia

## Abstract

**Background:**

Substance use among youths is a significant global and local issue. Youth who engage in substance use often experience various psychosocial, health, economic, and other problems. While governmental and non-governmental organizations in North Wollo and Waghimra Zones offer social services, their effectiveness in preventing and controlling substance use remains largely unstudied. This research aims to explore youth substance use prevention techniques and control mechanisms, youth participation, and potential challenges encountered by social service organizations.

**Methods:**

A qualitative case study design was employed in North Wollo and Waghimra Zones, Northeast Ethiopia. Purposive sampling was used to recruit participants. A total of 40 participants were involved, including 10 key informant interviews and 5 focus group discussions (FGDs) of youths (6 participants per group). The data collection instrument included a pre-tested semi-structured in-depth interview, an FGD guide, and an observation checklist. The collected data were analyzed thematically.

**Findings:**

Prevention efforts primarily focused on awareness-raising campaigns and advocacy programs. Control mechanisms were predominantly reactive, including measures such as regulating the substance trade, punishing drug dealers, destroying and burning drug paraphernalia, and closing substance trade centers. The study found limited youth involvement in substance use prevention and control initiatives. Several key challenges were identified in the prevention and control of youth substance use: limited government involvement, reliance only on reactive measures, low youth engagement, insufficient intersectoral collaboration, lack of community commitment, gaps in social institutions, absence of planned interventions, the economic role of substances, government ignorance, and the absence of comprehensive legal frameworks for prevention and control.

**Conclusion and recommendation:**

This study highlights a gap in prevention and control mechanisms, and engagement of youths, non-governmental as well as governmental bodies. This study recommends making evidence-based interventions and installing functional networks among sectors to deal with common issues. This issue also should not be left to a single organization and government agencies, families, religious, and educational institutions should play a leading role in tackling substance use burdens at the grassroots level.

## Introduction

Substance use problem is a pervasive global issue with varying prevalence and characteristics across different countries (1). It has become a significant public health concern, affecting individuals of all ages and socioeconomic backgrounds (2). The most used substances include tobacco, cannabis, khat, and alcohol (3).

In Ethiopia, substance use is a growing problem, with alcohol and khat being the most used substances (4, 5). These substances are deeply ingrained in the cultural fabric of many regions, making them widely available and socially acceptable. Tobacco is also frequently used, ranking third after alcohol and Khat (6).

Various factors contribute to the increased prevalence of substance use in Ethiopia (7). Urbanization and the increased availability of substances play significant roles. Additionally, globalization, unemployment, and social anomie are associated with substance use (8).

Adolescents are particularly vulnerable to substance use commonly with Khat chewing, alcoholism, and tobacco smoking (5–7, 9, 10). Substance use can lead to a range of health problems, including physical ailments, emotional distress, addiction, financial hardship, family breakdown, and social difficulties (11, 12).

Prevention and control efforts for substance use in Ethiopia are often inadequate. Studies have shown that weak prevention measures and a lack of prioritization for substance use issues are common (13). While some individuals may have the intention to quit, the availability of substances and peer pressure can make it challenging to overcome addiction (14).

Despite numerous studies on substance use being conducted in Ethiopia, most of them focused on its prevalence, associated factors, and consequences (4, 5, 8, 9, 15–19). However, there is limited evidence on the prevention and control mechanisms for substance use to our knowledge and through current research. Additionally, evidence indicates the presence of substance use youths in high school students in North Wollo Woldia City (20), and the study highlights socioeconomic and cultural factors contributing to the prevalence of such practices. These zones, located in the Amhara Region, are noted for agricultural activities that include Khat production, which is linked to local consumption patterns. Specific studies focusing on prevention and control mechanisms for youth substance use in North Wollo and Waghimra are scarce. Therefore, this study investigated the prevention and control mechanisms of substance use taken by a social service organization and came up with results important for developing intervention programs to address the problem. This study aimed to address the following objectives: (I) to explore the prevention mechanism of substance use; (II) to describe the control mechanisms of substance use; (III) to explore youths’ participation in the prevention and control of substance use; (Iv) to identify challenges in the prevention and control of substance use.

## Methods

### Study setting and design

A qualitative research approach was employed to gain in-depth insights into the prevention and control strategies for youth substance use in the North Wollo and Waghimra Zones. This methodology allowed for a holistic understanding of the issue from various perspectives, including the experiences and perceptions of the participants themselves. The study areas, North Wollo and Waghimra Zones, were purposefully chosen for two main reasons: first, their proximity and suitability for the investigators, which helped minimize costs and time constraints; and second, they are among the regions in Ethiopia with significant potential for substance use, particularly Khat.

A case study design using a phenomenological inductive qualitative approach was adopted for this research to enable an in-depth examination of substance use prevention and control efforts among youth-focused organizations in selected cities and towns because it helps to describe and explore the study issue in detail (21). The case is illustrated by multiple data sources such as focus group discussions (FGDs) with youths, in-depth interviews with key informants from various sectors involved in youth and substance use issues, and directly observing the issue. In addition, this study was bounded by place since it considers the prevention and control of substance abuse in selected areas only. Therefore, all these make this research a case study.

### Study participants and eligibility criteria

The study participants included youth, as well as government and non-governmental organization (NGO) officials from various sectors. Participants in in-depth interviews and FGD were identified with various criteria. Key informants for in-depth interviews were selected purposefully based on their willingness, position, experience, and relevance to the study. Specifically, the key informants were selected from youth associations; children, women, and youth affairs office; culture and tourism sector; health sectors; trade and industry office; education offices; and law enforcement offices. After the selection of key informants from these sectors, in-depth interviews were conducted with them. Youth participants were recruited based on their willingness, availability, and age (18 and above). Individuals who did not meet these criteria were excluded from the study.

### Sample size determination and sampling techniques

Data saturation was employed to ensure adequate data collection while maintaining a manageable sample size. This qualitative research principle involves continuing to collect data until no new insights are emerging (22). Based on this criterion, 10 interviews were conducted with key informants from service-providing organizations, and 5 FGDs were held with 6 participants each from relevant offices, to effectively address the study’s objectives. The observer was the data collector. The observation checklist was prepared in advance.

Eligible participants were selected using purposive sampling, a technique commonly employed in qualitative research. This approach involves deliberately choosing individuals who are likely to provide valuable insights into the study topic. This is because purposive sampling is widely used in qualitative research for the selection of information-rich cases related to the phenomenon, and criterion sampling is the most used purposive sampling technique. Moreover, the idea behind qualitative research is to identify participants purposefully based on the researcher’s judgment which helps to understand the problem and the research question. Criterion sampling, a specific type of purposive sampling, was used to identify participants based on their relevance and expertise. By carefully selecting participants in this manner, qualitative researchers can gain a deeper understanding of the research problem and related questions.

### Definition of terms

**Angada**: this is a relatively new substance utilized by civil servants in Ethiopia commonly in Afara and Kemissie.

**Kebele**: Kebele is the lowest administrative unit in Ethiopia. It’s roughly equivalent to a village or neighborhood.

**Substance use prevention techniques**: in this study, refers to strategies designed to reduce the initiation, escalation, and harmful consequences of substance use. These techniques aim to promote healthy behaviors, provide information, and address underlying risk factors that contribute to substance abuse.

**Substance uses controlling mechanisms and efforts**: in this study, substance use controlling mechanisms and efforts refer to the strategies and tactics employed to regulate and restrict the production, distribution, and consumption of substances. These efforts aim to reduce the availability of substances, deter illicit trade, and minimize the harmful consequences associated with substance abuse.

### Data collection instruments and procedures

Data were gathered through face-to-face individual in-depth interviews with key informants from various organizations, FGDs with youths in selected towns, and direct observations by data collectors. The data collection instrument is composed of four sections: (1) the demographic characteristics of study participants. (2) Semi-structured interview questions that were used to explore key informants’ experiences with substance use prevention and control mechanisms, the roles of their offices as well as their involvement and roles in these efforts, and the potential challenges for better prevention implementation. (3) The semi-structured FGD guides that were utilized to explore the youths’ participation and contribution to the prevention and control strategies of youth substance use. (4) The observational checklists used by the data collector to observe the actual practice related to practices of youth substance use and any involvements and strategies including training, public campaign, and advocacy practices and documentation in preventing and controlling youth substance use. Before the actual data collection, the data collection instrument was piloted and assessed for its clarity and content validity. After modification related to its clarity, the data collection was then conducted by experienced interviewers for qualitative studies. Audiorecods were applied during interviews and discussions. The key informant interview took an average of 31 min per interview and the FGD with youths took 65 min per FGD. The number of key-informant interviews and FGDs were determined based on data saturation.

### Data validation and verification

This study utilized different data validity and verification methods across different stages of the study. The data validity and verification measures applied in this study were employed during the data collection and analysis phases. To maintain credibility, we used triangulation of data sources and methods. Triangulation was applied by employing three different data collection instruments: key informant interviews, focus group discussions (FGDs), and an observational checklist. This allowed us to cross-verify information obtained from diverse perspectives, including key informants, youth participants, and direct observations of prevention practices. In addition, the research team engaged in regular discussions during data analysis to cross-check interpretations and ensure consistency in coding and theme development. This process minimized potential bias and enhanced the reliability of findings. Furthermore, we maintained detailed documentation of all steps in the research process, including data collection procedures, transcription protocols, and analysis decisions. This transparent process enhances the study’s dependability and allows for replication. Finally, data collection was continued until no new themes or insights emerged, ensuring that the dataset was comprehensive and representative of the participants’ perspectives. These methods ensured the quality and rigor of the study.

### Data synthesis

The data were synthesized using thematic analysis. The collected data were transcribed and organized for analysis. Findings from interviews, FGDs, and observations were triangulated to compare insights from individual interviews with group discussions and observational data to identify patterns or discrepancies requires a structured approach to effectively combine the qualitative data for comprehensive analysis. All collected data from interview transcripts, FGD notes, and observational records were analyzed to extract recurring themes. After reading the transcripts to identify key issues raised by participants, a coding process was conducted. This involved segmenting the text and assigning labels to relevant words, phrases, and sentences. Related codes were then combined to form themes, drawing on both the literature and the collected data. Each theme was subsequently analyzed in depth to produce thick descriptions. Finally, the findings were interpreted and presented. Participant quotations were presented with participant numbers to illustrate the findings.

## Results

### Demographic characteristics of participants

This study included a total of 40 participants (10 key informants and 30 FGD participants). The majority, 29 (72.5%) were male with a mean age of 36.1 (±8.2) years.

### Integration of main findings

The findings from the interviews, FGDs, and observations were integrated using a combination of triangulation, thematic synthesis, and comprehensive reporting. Triangulation was employed to cross-verify data, comparing individual insights from interviews, collective opinions from FGDs, and non-verbal or contextual cues from observations to ensure consistency and reliability. Through thematic synthesis, data from these sources were coded and analyzed to extract recurring themes, which were then mapped to show how different sources supported or added nuance to each finding. Finally, the integration was reflected in the reporting by illustrating themes with direct quotes from interviews and FGDs, supplemented by observational insights that added depth and context, creating a well-rounded and substantiated narrative.

### Common substances abused by youths

The study tried to explore the most abused substance by youths in the study area. As the finding indicated, the substance abuse by youth varies from town to town. Although their commonality is not uniform in place, khat, tobacco, shisha, alcohol, and Angada are found to be practiced in all towns of the study area. Our observation also supports this finding besides the data obtained from key informants and FGD participants. In some areas, khat is highly used and in other areas tobacco is highly smoked and in others, it all exists. Regarding the common substance used in the study area, one of the key informants (k1) spoke as follows:

“*Here in this town, youths are vulnerable to* var*ious substances. Since I do have experience living in this town for many years, I can mention what substances are highly utilized by youths including Khat”* (Youth follow-up and support officer from Kobo town Women Children and Youth office).

Based on the findings of this study, khat is one of the most abused substances among youth in the study areas. One of the key informants (k7) told the researcher his experience as follows:

“*I am working as a youth support and follow-up officer on behalf of Mersa town women, youth, and children office. Because of my work, I do have experience of contacting youths. It is not only because of my job but also because I know the town in detail. So as per my total experience, khat and shisha are the dominant substances abused here; but this does not mean there is no alcohol, tobacco, angada, and others. It seems that everybody is chewer because of the large number of chartbusters. The availability of khat-selling shops and chewing centers is a lot. If you go around the town, you will be surprised to what extent Khat is used here and there. Besides Khat, shisha is also extremely smoked in our town by youths. It is very common to sense the smile of shisha everywhere in the town. It becomes uncontrolled and beyond the capacity to protect it. Even civil servants also abuse Khat. Lots of them are absent from the office, especially in the afternoon chewing Khat. So, work time is also abused because of this. As a result, clients face* var*ious challenges in getting what they need from organizations. Youths justify unemployment, lack of recreation centers, peer influence, ease access of to two substances, and means of relaxation as their reason to enter such behavior. Youths also drink alcohol and smoke tobacco for further satisfaction. Therefore, youths are vulnerable to and experience multiple substance abuse. Unfortunately, we did nothing to address the problem since our department is at the commencing stage, which is a recently structured office. We do have a plan of working against the problem*.”

In support of the above findings, the researcher’s observation also confirms the existence of common substances in the study area. We have seen Khat users and traders, alcohol drinkers, and smokers in all study areas. Especially we have special experience regarding Khat in Woldia and Mersa towns. As per the researcher’s observation, some youths also smoke tobacco without preferring places though there is some kind of ban and restriction. Alcohol is also one of the observed things in all study areas which is mostly practiced in the afternoon and at night. There is also a place where alcohol is drunk without the preference of time from morning to night at Kobo town.

### Prevention mechanisms and efforts of substance use among youths

The study identified common substances used by young people in the study area, including khat, tobacco, shisha, alcohol, and angada. Data collected from key informants and focus group participants revealed that substance use prevention mechanisms primarily focus on awareness-raising and advocacy programs.

### Awareness-raising and advocacy programs

The study found that limited efforts to address youth substance use are being undertaken by government institutions. However, awareness creation prevention activities are often integrated with other programs, and existing organizations may not be fully utilizing their capacity to address substance use issues. Furthermore, the study identified a significant gap in NGO involvement, with no single organization focusing solely on this problem.

Concerning prevention efforts, one of the key informants who work with youth-related institutions (k1) reported as:

“*I am working at women’s children and youth office as a youth support and follow-up officer. Our institution provides* var*ious services for youths and others. Among these services, substance use prevention can be mentioned as an instance. Regarding this, our main task is facilitating and providing awareness-raising training about the negative impacts of substance use. We have youth support officers at a ‘kebele’ level. Therefore, we access this training for youths. This is because some youths have a gap of awareness on this issue. They use substances unknowingly of their consequences. However, some youths engage in this hurtful activity knowingly due to* var*ious reasons such as having fun, hopelessness, lack of a job, peer influence, avoiding stress, and others. Anyways, as a concerned body, we provide awareness-raising training for those who are willing to participate. To provide this training, we collect training manuals at regional and zonal offices. The training is delivered at ‘kebele’ centers. This is the usual activity that we do to save our youths at our office level. But I do not believe that it is enough to address the problem*.”

In support of the above description, the other key informant from the zone police department (K4) stated their concern on the prevention of substance use as follows:

“*I do have an experience of long years as police in different positions at* var*ious woredas of North Wollo Zone. Currently, I am working at the zonal office. North Wollo zone is highly vulnerable to substance use. Youths are the main victims of this problem. If you travel around North Wollo, there is no khat and alcohol-free town. The productive age group of our population is seriously damaged by this problem. As our office, our main concern is preventing criminal activities and securing peaceful conditions in the towns. This does not mean we are not concerned about youth life. This is because we work on the prevention of substance use and narcotic drugs. We are aware of youths about substance use while performing our regular crime prevention activities. Therefore, what we are doing is providing some lessons on substance use combined with* var*ious crime prevention education. Therefore, there is no independent substance use prevention program, but merging with other activities. The reason why we engage in the prevention of substance use is because addiction is the cause of* var*ious crimes. When people get addicted to substances, they engage in theft, hang, rap, and other crimes. Therefore, to prevent such crimes, we are aware of the youths about the multiple effects of substance use collaboratively with our community policing wing. We also invite health experts, especially health extension workers, to cover the health aspects of substance use during the learning sessions.*

Awareness raising as a mechanism of substance use prevention was also practiced by culture and tourism offices. One of the key informants from this office reported that they engage in facilitating awareness-creation programs to alleviate substance use problems in the town. This awareness-raising program is accomplished using different techniques. The key informant (K2) stated their office experience in the following ways:

“*I am the head of the culture and tourism office. Mainly, we work on the issue of culture and tourism. But we also work on youths since they are our concerns. Practically, we are involved in preventing substance use among young people through creating awareness. We try to educate youth about the multi-dimensional impact of substance use mainly through arts. We arrange* var*ious arts such as drama, theater, music, and poem time. We invite youths to attend this program and transfer messages about this problem in this way. This methodology has a dual purpose, entertainment and teaching. There are two art and culture clubs established and managed by our offices. These clubs have different roles including fighting substance use and other social problems. They prepare shows at ‘kebele’ Center, our centers, and different halls. The program is opened for all youths freely since it is intentionally organized to aware youth and the community at all through the art department.*”

Another participant (K3) described their organization’s experience as follows:

“*To prevent youth substance use, we organize and facilitate awareness-raising sessions. The awareness solely focuses on multidimensional short and long-term consequences and impacts of substance use. This awareness is accompanied using events like African and World Youth Days. These days, a message is transferred to youths to protect themselves from substance use. Awareness is also reached to youths on behalf of different youth-focused associations. We create awareness about substance use at places where collected people are there, such as bus stations and others. We were also aware of youths through preparing training documents and distributing fliers and leaflets*.”

Regarding awareness creation as a prevention mechanism of substance use, it is also accompanied by the Health Office. Related to this, one of the study participants (K5) reported that “*the health office creates awareness for youths to understand the health impacts of the problem. The awareness is done through peer-to-peer training, posting posters, distributing leaflets, and home-to-home rounding relating to HIV/AIDS. The awareness is prepared for youths within the community and commercial sex workers. Our office was also aware merchants not to sell tobacco per piece but by packet.”*

Moreover, the other study participant (K6) indicated that “*awareness raising is provided for youths especially for students at school through mini media clubs. However, this activity cannot be mentioned as exemplary practice; and no NGO is working on this issue at schools. Mini-media and other clubs have great potential to make students aware of this problem but are not fully functional as well.”*

One of the study participants (K10) who works at non-governmental organizations (NGOs) mentioned that “youths *are their concern, but their project focused on reproductive health. They sometimes organize and conduct workshops on addiction to aware youths. However, the organization has not had a trend of evaluating the impact of the workshop. And totally, they are not concerned deeply about substance use except by facilitating some workshops which cannot be stated as best practices.*

### Advocacy programs

According to this study, officers at different organizations also engaged in preventing substance use among youths through playing an advocacy role. They speak on behalf of youths who are their direct clients. They tried to save youths from addiction by struggling for them on different occasions; especially concerning the access to job opportunities and youth recreational centers. Concerning this, one of the study participants (k1) reported what he has done so far as follows:

“*I am a youth support and follow-up officer at my organization. This position obligates me to provide* var*ious services for youths since they are the direct beneficiaries of the position. One of the expected services is saving youths from addiction. To meet this expectation, I provide an advocacy service besides awareness creation. When you interview youth about why they engage in substance use, they respond due to unemployment, and the absence of youth centers and recreation areas. So, I speak representing them at different meetings with officials as I am their servant. I reflect on their voice, needs, and problems here. I challenge officials to use any opportunities to access jobs for youths and to build youth centers. This is because if youths are busy with* var*ious income-generating activities, they will not have time to go to addiction behaviors. Furthermore, youths can also spend their free time at recreational centers instead of using substances. To this end, I play advocacy roles as per my capacity and opportunities allowed; but I do not believe that this is enough to protect youths from addiction. It is only women, children, and youth affairs that attempt to address the problem; and there are no supporting NGOs on substance issues in our case.”*

In support of the above description, the other key informant from the youth association (k8) stated how he advocates for youths as cited below:

“*I am the president of the youth association. All my responsibility is securing the benefit of youths. We work for the creation and development of healthy, productive, and visionary youths in our town particularly and at the country’s level generally. If you see the government, he neglects the issue of youth. It seems that the Government does not bother about the lives of youth. It only worries about its political power. Youths are blocked from participating in* var*ious development issues and fruits. They are simply paralyzed and simple observers of the beneficiaries of systems. There are large numbers of unemployed youths in our town. They engaged in* var*ious substance use behaviors since there was no other opportunity. Therefore, as youth representatives, we do not let them be silent when they are damaged and become useless. We struggle for them even if the association is at its infant stage. We communicate and debate with government officials to make youth active participants and beneficiaries of development issues. We advocate for young people to be organized and involved in business. Besides this, we also raise youth issues at different meetings of officials to create youth centers, to expand and facilitate sports areas. We also try to pressure the government to make youths his concern.*”

### Substance use control mechanisms and efforts

This study revealed that most control mechanisms were reactive measures after the events happened rather than preplanned and organized systems. Concerning control efforts of substance use, one of the key informants (k4) from the police office said the following:

“*Accordingly, our department tries to control some substance-abusive behaviors. In this regard, we control Shisha smoking centers when we get information. Shisha is mostly unobservable. It is utilized illegally at illegal centers. It is hidden smoke and difficult to detect. Most of the time, there is shisha behind bars. So, we study shisha smoking areas and when we find it, we will burn to collect the materials. People who engage in this business will be treated by local social courts. They will be punished financially. They were also advised by the sector not to repeat this action and not to hurt their people. The houses open for this service are subject to closure because somehow, they are against the societal norm and without a trade license. However, we cannot control khat chewing, alcoholism, and angada. Because there is no legal restriction against such misbehaving. There is a wide legal gap in this regard. Due to this reason, our role in controlling the issue is weak. There is no strong mechanism that can be described as the best example. Even society is not collaborative in indicating some illegal behaviors that cause crime. We have also a gap in controlling tobacco though there is some implementable legislation.*”

The study participant (k5) from the health office agreed with the above narration and mentioned that “*we control shisha smoking collaboratively with the police office. We also try to stop the entrance of shisha smoking materials at Kela collaboratively with the revenue office. We control any promotion of alcohol in the city and control the single sale of tobacco rounding shops and trade centers. If we get someone who sells tobacco per peace punishment will follow that extends up to canceling trade lice and closing trade centers. However, we have failed to stop tobacco smoking in public areas although a clear legal framework is available. Nobody has been punished so far due to the violation of this ban. The only thing we did to control this behavior was post smoking restriction posters at hotels and public areas where collected people are there.*”

Another participant of the study selected from school (k6) reported that “*we control substance use in the school through implementing the school rules and regulations. Based on school guidelines, smoking tobacco and chewing khat inside the school compound is strictly forbidden. A student who violates school guidelines is subject to punishment like rejection from the school for a year. Also, the student is requested to bring his/her parents and para-counseling service will be provided.”*

Another study participant (K3) added “*We attempted to burn shisha smoking materials and block the houses open for this purpose, but it is seasonal and not sustainable work. This kind of doing is performed in certain seasons but not repeated and extended. Once it is tried to control the problem in a campaign; it is never repeated*.”

Another key informant from trade and transport (k9) indicated that ‘*we are not responsible for controlling substance use. Our only role is controlling people who engage in substance trade without a trade license. We punish those without a license by blocking their shop/center to arrest by police. We collaborated with police while taking punishment illegal people engaged in substance selling*.”

### Youth’s participation in substance use prevention and control

In this study, it is found that the participation of youth to combat substance use is not encouraging. Youths miss various awareness creation programs organized by service providers. They are reluctant and hopeless in finding solutions through meetings and training.

Regarding this, one of the key informants (k1) described the participation of youths as follows:

“*As concerned with youth life, we facilitate and arrange awareness creation programs about substance use. We try to disseminate the message and call youths to come to us through notice and rounding the ‘kebeles’. Although we try some, most youths do not attend. Only a small number of youths participate in pieces of training. This makes our efforts fruitless. I blame youths in this regard. They must participate actively because it is for their own life. They should contribute their effort for themselves.”*

On the other hand, the other key informant of this study (K2) reported the participation of youth as follows: “*Truly speaking, our office’s efforts in the prevention and control of youth substance use are minimal. Though it is not sufficient, we have made some attempts. We try to organize awareness-raising and educational programs through our cultural clubs. Using these clubs is an opportunity for alerting youths about the negative impact of substance use through art. There are different dramas and artistic periods. We transfer messages through speaking, distributing fliers, and using posters. But we did not get enough number of participants as we expected. The attendants are very low in number. Youths do not have a positive attitude toward such kind of program. So, what I advise is that youths should be attracted to these programs. They will not lose anything instead of benefiting from participating here. It is for their sake that we access these programs. So, it is better not to miss it.”*

Another participant (k5) reported that “*some youths have positive attitudes, and some youths do not have any information about substance use. Some others are reluctant to participate in preventing and controlling the problem, and they correlate with political agenda and dislike participating in this process.”*

Youths from the FGD participants told the researcher the following:

“*No one wants to engage in substance use intentionally. It is a situation that obliges and leads to the development of this bad habit. There is no job; there is no healthy place to spend time. So, what shall youths do?* Var*ious other factors push us to engage in this behavior. If things become safe, addicted youth also can stop this behavior. Because they know that they are losing their life and killing themselves. They are intended to exit from this behavior if they get support in different dimensions. We are committed enough to contribute to preventing and controlling substance use. I can say that the government’s effort in this regard is zero. I think the government also needs youth addiction; because if youth become weak due to addiction, no one struggles against the government. It governs the country as it needs without powerful competition. Youths also become bored enough by a series of meetings. Meetings cannot solve the youth’s problem. We need some tangible support that can change our lives. Even though we organize ourselves to engage in business, nobody supports us financially. If the government is committed, youths will not waste their time by abusing substances. Therefore, we need the support and attention of the government strongly.”*

In line with this, other participants in the FGD session reported that “*we were involved in some drug-related illegal activities such as Shisha bets. We are also aware of and share their experience regarding addiction with their fellow youths. we also showed their commitment to sharing their lived experience of substance use if the concerned body facilitates and organizes events*.”

Concerning youth participation in the prevention and control of substance use, one of the study participants (k3) strengthened the above narration and reported “*We are supportive of the control and prevention process. Youths have negative attitudes towards substance use, but they need tangible and life-changing support*. As reported by k5 youths also fail to engage in business based on their license. They rent the container that the government accessed for disallowed business out of their license.

### Challenges to the prevention and control of substance use

Several key challenges were identified in the prevention and control of youth substance use: Limited collaboration, limited government involvement, reliance only on reactive measures, low youth engagement, lack of community commitment, gaps in social institutions such as families, religion, and education, absence of planned interventions, the economic role of substances, government ignorance, and the absence of comprehensive legal frameworks for prevention and control.

### Collaboration problem

Based on this study, the partnership problem is found to be one of the challenges in preventing and controlling substance use. There is no strong network which is installed against this problem. Even if some networks are available, they are not functional and effective as expected. There is not a culture of collaboration for common issues. Concerning this, one of the key informants (K1) described this challenge as follows:

“*There is no collaboration of concerned bodies in fighting substance use. If you ask some sectors, they reply that it is not their concern. If you ask others they respond similarly. In the middle, youths are highly damaged. There is no strong partnership for common issues. There is no strong connection among sectors for the achievement of common goals. Police try their own. The health sector tries its own. Women children and youth affairs and culture and tourism also make some attempts individually. But the problem is deep; it needs multi-sector collaboration. If they work together, encouraging change can be brought up. Otherwise, it is tough to address the problem.”*

As reported by k5, the inter-sectorial linkage is found to be an irresistible challenge. He elaborated that “if *the health sector performs the awareness, small and micro-enterprises are expected to organize youth to engage in business; and financial institutions should grant the funds for youths. Concerning the absence of collaboration as a challenge, most of the key informants agreed with K1 and k6 and mentioned it as a big problem. They indicated that it would be better if they could work collaboratively to address all youth issues.”*

### Government’s neglecting

The key informants from government workers reported that the researcher that “*higher government officials are not worried about youth life*. *Nobody cares for the youth’s life. Besides this, the government also does not allocate a budget to manage this problem*. *There is not any single NGO that works on this issue and fills the government gap in this regard.”*

Regarding the government’s reluctance to allocate a budget to address this problem, one of the study participants (k3) told the researcher the following:

“*As per my experience at my office, there is not any budget allocated to fight youth substance use. I can say that there is no budget for this problem at all. The government ignored this issue, but the reality shows that it needs serious concern. As youth-concerned workers, we have presented the issue of budgeting and planning for officials, but still, there is no change in this regard. Even rehabilitation centers to treat addicted youth are highly needed, but nothing can be done without an adequate budget. What we are doing is in parallel with other budgeted activities*.”

### Low community participation

The study indicates the presence of gaps in community participation in the prevention and control of substance use. Key informants agreed and said “*The former strong community’s norm is becoming weak and eroded from time to time. Although the community opposes youth substance use, it is reluctant to shape youths and collaborate with others to fight substance use.”*

As key informant (k4) stated “*Some people from the community engaged in renting their house for shisha, angada, and other dangerous drugs. Hence, they are cooperative substance mobilizers.”*

Participants of the study (K1, k2, k4, and k8) indicated that “*the family’s responsibilities in socializing their children are not to engage in substance use and to respect societal norms and taboos are low. It is also shown that religious institutions are not responding to their responsibility to the greatest extent in producing ethical and drug-free youths and generations*.” In addition to this, K5 indicated that “*the community should be concerned about substance use among youths. It is needed to develop a feeling of ownership of the case. People should not be silent when somebody smokes nearer to them since it makes them passive smokers and harms their health. The community also participates with institutions in the control and prevention process of the problem.”* K1 recommends that “*Woldia University make the community* via *its FM radio to be active participants in addressing this problem.”*

### Absence of a strong legal framework

This study showed that there is no clear legal framework to control the common substance in the study area. As per this study, substance use has become a culture among youths where policymakers and law developers left the issue aside thinking that it would have no solution. As cited by a key informant (k4): “*khat chewing is not forbidden by law. It is a legal product that is sold and chewed in every corner of the town. Hence, it is impossible to protect and take legal measures against those chewers as well as traders of this substance. It is difficult to take strong measures against shisha users and those who engage in this business. This is because there is no legal ground to refer to the behavior as a crime. The only controlling measurement is punishing those actors who engage in substance-related trade without legal trade licenses.”* Other key informants of this study from health, youth associations, and children, women, and youth affairs office agree with the above description from the police office.

### Substance as a source of income and employment opportunity

As key informants of the study indicated “*several farmers engaged in cultivating khat to lead their lives. The government also collects a large amount of income from exporting khat*. Most of the key informants agreed that “*khat is a business for many youths and serves as a means of income for traders at a local market*. *Substance-related trade is considered a big business and employment opportunity for youths.”*

Another informant reported that “*The city and town administration also collect large amounts of tax from substance trade, especially from khat. It is too difficult to stop these youths from engaging in this business since they are the beneficiaries of the problem. There must be another alternative business idea that helps these youths to address the problem (k5).*

Most of the key informants also told the researcher that “*it is essential to convince farmers to stop cultivating khat and replace it with other healthy and profitable fruits and farming activities; otherwise, the problem will worsen. Therefore, this is the biggest blockage and bottleneck to prevent and control the problem*.

### Absence of planned intervention

Based on this study, it is found that the absence of planned intervention to prevent and control substance use is a problem. Concerning this challenge, k3 clarifies his concern as follows:

“*Substance use among youth is visible in our towns. As far as my knowledge and experience are concerned, there are no planned interventions, programs, or projects implemented by the government or NGO to address the problem. The problem needs evidence-based intervention unless it is impossible to manage it. The GO and NGOs should engage in curving this problem scientifically. The existing experience of our office related to this issue is seasonal, inconsistent, and unplanned. We try to prevent and control the problem in a team at a point in time and forget it after all. However, the problem cannot be avoided in this way; it needs organized, planned, intentional, and regular long-term effort*.” The other key informants of the study (k1, K2, k4, k7, and k8) agree with a key informant (k3). They indicated that their experience in this regard is also unplanned and timely.

## Discussion

This study examined prevention and control strategies implemented by government and non-governmental organizations, youth participation in these efforts, and the primary challenges in addressing substance use among youths. Prevention mechanisms for commonly used substances such as khat, alcohol, shisha, tobacco (5–8, 10), and angada primarily focus on awareness-raising and advocacy. These findings align with other studies (13, 23, 24). Some sectors provide education on the harmful effects of substance use through training, public events, and materials distribution. Others advocate for youth’s needs and raise awareness among officials.

However, the study found that prevention efforts for youth substance use are inconsistent and have not received sufficient attention. There is a lack of independent, well-funded, and structured intervention programs from government and non-governmental organizations in the study area. Existing efforts are fragmented and integrated with other services, limiting their effectiveness in reaching at-risk youth. To enhance prevention and control, utilizing mass media for educational programs is recommended (25). Mass media can be a valuable resource for educating and alerting young people about the negative consequences of substance use. Despite the importance of awareness-raising, the study observed limited involvement from youth, government sectors, and supporting NGOs. Leveraging various options such as public meetings, events, training, fliers, arts, mini media, peer-to-peer training, and home-based activities can be effective in raising awareness.

Regarding control measures for youth substance use, consistent with other research (26, 27), concerned sectors have implemented measures such as punishing illegal substance merchants, closing trade centers, and imposing fines. While efforts by police, health, trade, and education offices have been made, respondents perceive these measures as insufficient to address the severity of the problem. Despite the rapidly increasing prevalence of substance use, there is a lack of robust and consistent control systems. Existing control mechanisms are largely reactive, including measures such as regulating substance trade, punishing drug dealers, destroying drug paraphernalia, closing substance trade centers, burning shisha materials, imposing financial penalties through local social courts, canceling trade licenses, closing shops of illegal traders, and controlling alcohol promotion. These measures are often temporary and unsustainable. Overall, the findings highlight the need for a strong legal framework, effective control mechanisms, and community involvement in addressing substance use.

The study found that youth participation in prevention efforts is limited, with many viewing awareness programs as government propaganda. However, other research has demonstrated the significant potential of youth involvement in substance use prevention (28–30). While some youth support substance uses prevention and control efforts, others remain disengaged. The study suggests that youth are more interested in tangible support than in awareness-raising, as they are already well-informed about the issue. Some youth actively participate by reporting illegal drug activities and sharing their experiences with peers. However, their involvement is often unstructured and lacks organizational support. The findings emphasize the need to strengthen youth engagement in substance use prevention and control policies and frameworks.

Consistent with previous research (14), this study identified several challenges in substance use prevention, including weak intersectoral partnerships, government neglect, low community engagement, and the cultural normalization of substance use. The study found that the government has shown limited attention to youth substance use, often overlooking the problem. Substance use has become a source of income for youth, merchants, and farmers, making it difficult to address (31, 32). The government’s involvement in exporting certain substances, such as khat, further complicates the issue. This study suggests breaking the cycle of using substances as a source of income and employment. Farmers should also be trained to discontinue khat cultivation and explore alternative, healthy crops.

The study found a lack of dedicated organizations focused on youth substance use problems, aligning with previous research (14). Existing organizations, such as women’s, children’s, and youth affairs offices, police, culture and tourism departments, youth associations, education, and health sectors are unevenly distributed across study sites. While nominally involved, these organizations often prioritize other issues and lack specific plans or programs for substance use prevention. As a result, the response to youth substance use is fragmented and lacks a coordinated approach. The study suggests that NGOs should implement evidence-based interventions that directly benefit youth, given the absence of such organizations in the study sites. The pressing nature of substance use issues makes it an attractive area for NGOs to implement projects that can positively impact youth and the broader community. The study also highlights the potential role of religious institutions in promoting ethical behavior and adherence to religious principles and community norms among their followers.

Addressing youth substance use requires a collective effort. It is not solely the responsibility of women, children, and youth affairs offices. As a generational issue, all institutions should be involved. Families should foster healthy development and social behavior among their children and youth, aligning with community norms. Family-based interventions are crucial for raising resilient young people. Educational institutions should integrate substance use education into their curricula to contribute to the development of productive and healthy future generations.

The government should establish strong legal frameworks and ban commonly used substances in the study area. As participants noted, legal measures can be effective in preventing and controlling substance use behavior. Without clear and understandable legal frameworks, it is impossible to hold accountable those who engage in substance use and harm themselves or others. Innocent individuals are also exposed to various harms due to the actions of substance users. Substance use among youth should be prioritized, and sectors should develop and implement evidence-based plans and projects. Relevant structures should be organized, and qualified personnel should be assigned to address youth substance use issues.

A strong task force should be established to address substance use. Sector organizations concerned with youth issues should collaborate effectively, as individual efforts are unlikely to be successful. A multi-agency approach is essential to manage this problem. Inter-sectoral linkages must be strengthened and culturally sensitive. Policy and legislative advocacy are needed to implement existing measures at the grassroots level.

### Study’s strengths and limitations

The current study has important strengths. Addresses a Critical Gap: The study fills an important gap by focusing on prevention and control mechanisms, whereas much of the existing research has been limited to substance abuse prevalence and associated factors in Ethiopia.

Use of multiple data sources: The triangulation of FGDs, in-depth interviews, and observations strengthens the study by providing a comprehensive view and ensuring richer, more reliable data.

Thematic analysis: The use of thematic analysis with a systematic approach (coding, theme development, interpretation) enhances the study’s rigor and transparency, which increases the reliability of the findings.

Community and youth focus: By highlighting youth participation and community engagement, the study identifies actionable areas for improving substance abuse prevention, making it relevant for policy recommendations.

However, this study has some limitations. Limited generalizability: Being a qualitative case study focused on specific social service organizations in North Wollo and Waghimra Zones, the findings may not be generalizable to all regions of Ethiopia or other settings. This geographical limitation restricts the applicability of the results to broader populations.

Potential sampling bias: Although purposive sampling is appropriate for qualitative studies, it can introduce bias, as participants are chosen based on the researcher’s judgment. This selection may overlook perspectives from other potentially relevant stakeholders or youth not engaged with social service organizations.

Lack of quantitative data: The study does not incorporate quantitative data, which could have complemented the qualitative findings and provided a broader scope of insight, such as measuring the actual impact of existing prevention and control measures.

Reliance on self-reported data: As with most qualitative studies, the reliance on self-reported data from interviews and FGDs introduces the possibility of social desirability bias, where participants may present their organizations or actions in a favorable light, possibly overlooking critical challenges or shortcomings.

Focus on organizational efforts only: By primarily examining social service organizations, the study may not capture the full scope of substance abuse prevention efforts, particularly informal strategies or interventions by family, religious groups, or peer networks, which are also influential in substance abuse prevention.

Future studies should address the following points for further improvement.

Expand geographical scope: Including multiple regions could improve the generalizability of the findings and provide a comparative perspective.

Incorporate quantitative elements: Collecting some quantitative data on the effectiveness of specific prevention and control mechanisms could provide additional insights, offering a more holistic view.

Include more diverse stakeholders: Expanding participant criteria to include other influential groups, such as religious leaders, family members, or peers, could provide a more comprehensive understanding of the community’s role in substance abuse prevention.

## Conclusion

The study has revealed prevention and control mechanisms and youths’ participation in tackling substance use is limited. Different challenges extending from low community and youth involvement and weak government commitment to prevent and control youth substance use were observed in this study. The study highlights the need for evidence-based interventions and installing functional networks among sectors to deal with common issues. This issue also should not be left to a single organization and government agencies, families, religious, and educational institutions should play a leading role in tackling substance use burdens at the grassroots level.

## Data Availability

The original contributions presented in the study are included in the article/supplementary material, further inquiries can be directed to the corresponding author/s.
